# FITC-Labeled Alendronate as an In Vivo Bone pH Sensor

**DOI:** 10.1155/2020/4012194

**Published:** 2020-05-19

**Authors:** Yuzhou Li, Yiru Fu, He Zhang, Jinlin Song, Sheng Yang

**Affiliations:** ^1^College of Stomatology, Chongqing Medical University, Chongqing 401147, China; ^2^Chongqing Key Laboratory of Oral Diseases and Biomedical Sciences, Chongqing 401147, China; ^3^Chongqing Municipal Key Laboratory of Oral Biomedical Engineering of Higher Education, Chongqing 401147, China

## Abstract

pH is a critical indicator of bone physiological function and disease status; however, noninvasive and real-time sensing of bone pH in vivo has been a challenge. Here, we synthesized a bone pH sensor by labeling alendronate with the H+-sensitive dye fluorescein isothiocyanate (Aln-FITC). Aln-FITC showed selective affinity for hydroxyapatite (HAp) rather than other calcium materials. An in vivo biodistribution study showed that Aln-FITC can be rapidly and specifically delivered to rat bones after caudal vein injection, and the fluorescence lasted for at least 12 h. The fluorescence intensity of Aln-FITC binding to HAp linearly decreased when the pH changed from 6 to 12. This finding was further confirmed on bone blocks and perfused bone when the pH changed from 6.8 to 7.4, indicating unique pH-responsive characteristics in the bone microenvironment. Aln-FITC was then preliminarily applied to evaluate the changes in bone pH in a nude mouse acidosis model. Our results demonstrated that Aln-FITC might have the potential for minimally invasive and real-time in vivo bone pH sensing in preclinical studies of bone healing, metabolism, and cancer mechanisms.

## 1. Introduction

pH plays an important role in bone homeostasis [1–4], and disruption of bone pH is associated with tumors, inflammation, fractures, and hypoxia [1, 2, 4, 5]. Real-time sensing of bone pH can provide valuable information for diagnosis and research, but delivery of the sensor is challenged by hard and solid cortical bone [6]. Previous studies have tried to insert a pin-like microelectrode into the bone, which could report the pH value through a wired, external recorder [7, 8]. Recently, a pH-responsive hydrogel-based implanted sensor with a metal pin was developed, where the pH data could be collected by a series of X-rays [9]. However, these sensors were all constrained by their expensive devices, low spatial resolution, and invasive surgery. To overcome these limitations, we assumed that a good bone pH sensor should be easy to fabricate, easy to deliver, and sensitive to real-time pH changes.

Fluorescent dyes are widely used in biology to sense organic targets such as proteins [10, 11] and nucleotides [12] and inorganic targets such as calcium [13], sodium [14], and hydrogen ions [15, 16]. Among these dyes, fluorescein isothiocyanate (FITC) has a H+-dependent emission spectrum over the range of pH 5-9; thus, it is widely applied to sense the pH in biological conditions such as cells, soft tissues, and body fluids [17, 18]. However, whether FITC can sense real-time changes in bone pH has not yet been studied.

The effective delivery of fluorescent sensors to the bone and maintenance of its concentration can also be challenging. Since the bone mainly comprises hydroxyapatite (HAp), molecules can be specifically targeted to the bone through modification with HAp affinity ligands, such as polyamino acids [19, 20], polypeptides [21], and phosphonates [22]. Alendronate, a typical phosphonate, can tightly bind to HAp through its two phosphate groups and has been broadly used in bone-targeting vehicles [23] and drugs [24]. In addition, alendronate can be retained in the bone for a long period of time [22]. Hence, modifying sensors with alendronate could reasonably enhance targeted delivery to the bone.

Herein, we introduce a bone pH sensor synthesized by alendronate and FITC, namely, Aln-FITC. The high reactivity of the isothiocyanate group (FITC) and amino group (alendronate) allowed the synthetic procedure to be simple and fast. The biocompatibility, biodistribution, and pH-responsive fluorescent characteristics of Aln-FITC were evaluated. The pH-sensing ability of Aln-FITC was primarily tested in a metabolic acidosis animal model. To our knowledge, there have been few efforts regarding the use of fluorescent dyes to sense bone pH in vivo. We hypothesized that Aln-FITC can work as a simple, safe, and effective bone pH sensor with future potential in preclinical studies for the in-depth understanding of changes in the bone microenvironment during healing, metabolic disease, and cancer development.

## 2. Materials and Methods

### 2.1. Ethics Statement

All experiments and procedures were performed in accordance with the appropriate guidelines of Chongqing Medical University. All operations for animals followed the guidelines of the Institutional Animal Care and Use Committee of China and were approved by the ethics committee of Chongqing Medical University Affiliated School and Hospital of Stomatology (2019073).

### 2.2. Synthesis of Aln-FITC

Fluorescein isothiocyanate isomer I (1.13 *μ*mol, F7250, Sigma) was dissolved in DMSO and then mixed with 11.3 *μ*mol of alendronate dissolved in bicarbonate buffer, pH 9.0. The volume was diluted to 1 ml with distilled water, and the solution was incubated for 2 h at room temperature under constant mixing. To precipitate alendronate, 39.6 *μ*mol of CaCl_2_ was added, and the mixture was centrifuged (14,000 g, 10 min). The precipitate was washed with 1 ml of distilled water five times. To redissolve alendronate, 39.6 *μ*mol of EDTA was added to the precipitate. Then, 100 *μ*l of PBS was added until all precipitate dissolved, and the solution was shaken with ultrasonication for 30 min. The final solution (referred to as Aln-FITC) was lyophilized, and an orange powder was obtained containing FITC-labeled alendronate and free alendronate, which was quantified and purified by liquid chromatography.

### 2.3. Characterization of Aln-FITC

Mass spectrometry (MS) spectra were obtained with 100% acetonitrile containing 0.1% formic acid in water at a flow rate of 0.3 ml/min using UP LC-MS (Shimadzu LC-MS 8060). Fourier transform infrared (FTIR) spectra were obtained between 400 and 4000 cm^−1^ with a resolution of 4 cm^−1^ using a Nicolet iS50 spectrometer (Thermo Fisher Scientific, USA) with the KBr disk method.

The absorption, excitation, and emission spectra of FITC, alendronate, and Aln-FITC were measured using a multiplate reader (EnSpire, USA). The absorption spectra were measured at wavelengths between 300 nm and 700 nm. For the excitation spectra, samples were excited from 300 to 500 nm with a 10 nm step and measured at 530 nm. For the emission spectra, samples were excited at 480 nm and measured in the range of 500 to 700 nm with a 10 nm step.

### 2.4. Cell Culture and Cytotoxicity Test

To acquire bone marrow stromal cells derived from rats (rBMSCs), two 6- to 8-week-old male Sprague Dawley (SD) rats were sacrificed by cervical dislocation, and their femurs and tibias were carefully cleaned from the adherent soft tissue. The tip of each bone was removed with a rongeur, and the marrow was harvested by inserting a syringe needle (27 gauge) into one end of the bone and flushing with Dulbecco's modified Eagle's medium (DMEM, Gibco). The bone marrow cells were filtered through a 70 mm nylon mesh filter (BD Falcon, USA). Cells were plated into dishes (*d* = 10 cm) in DMEM containing 15% FBS, 100 U/ml penicillin, and 100 U/ml streptomycin. Cultures were kept at 37°C in a humidified atmosphere containing 95% air and 5% CO_2_. When the primary culture reached 90% confluency, the cells were treated with trypsin (0.25% *w*/*v*) and EDTA (0.02% *w*/*v*) for 2 min at room temperature and then subcultured in new dishes.

rBMSCs were cultured in 96-well plates (5,000 cells/well) for 24 h and treated with Aln-FITC over a range of concentrations (0 as the control, 0.1, 0.5, 1, 5, and 10 *μ*M). After 24 h, the rBMSCs were thoroughly washed to remove excess Aln-FITC, and cell growth medium (100 *μ*l/well) containing CCK-8 solution (10 *μ*l/well, MCE) was added to each well, followed by incubation for 4 h at 37°C. Then, the medium was transferred to a new 96-well plate to avoid any interference from intracellular Aln-FITC, and the absorbance at 450 nm was measured by a multiplate reader. For each concentration of Aln-FITC, the mean value was calculated from six samples.

### 2.5. In Vitro HAp and Bone Affinity of Aln-FITC

Aqueous suspensions of calcium species (HAp, CaSO_4_, and CaC_2_O_4_; all purchased from Macklin, China) (5 mg/ml) were incubated with Aln-FITC (1 *μ*M) for 2 h. The unbound sensor in the supernatant was removed through centrifugation at 600 g for 5 min, and the precipitate was washed with water under continuous agitation for 15 min. This process of centrifugation and washing was repeated three times. Fluorescence analysis of the final aqueous product was performed using fluorescence microscopy.

Passage 4 rBMSCs plated at 10,000 cells/well in a 6-well plate were incubated in DMEM supplemented with 10% FBS and 100 U/ml penicillin/100 U/ml streptomycin for 2 days. Then, the proliferation medium was replaced with osteogenic medium containing DMEM with 50 *μ*M ascorbic acid 2-phosphate (Sigma), 10 nM dexamethasone (Sigma), and 10 mM *β*-glycerol phosphate (Sigma). The cultures were then placed in an incubator at 37°C and 5% CO_2_ for 21 days with media changes three times per week. At the end of the cultivation period, the media was removed, and the cell monolayer was washed with PBS and then incubated with DMEM containing Aln-FITC (1 *μ*M) for 2 h. The media was removed and the cell monolayer was thoroughly washed three times with PBS, fixed with 4% PFA for 15 min, and washed with PBS (3x). The cells were then stained with Alizarin S (2%, pH 4.2) for 5 min at room temperature. The supernatant was discarded, and the cell monolayer was washed with PBS (3x) and then imaged at excitation wavelengths of 530 nm (Alizarin S) and 488 nm (Aln-FITC).

### 2.6. In Vivo Bone Affinity and Toxicology of Aln-FITC

Aln-FITC was intravenously injected into 3 male SD rats through the caudal vein at a dose of 8 *μ*mol/kg body weight. The rats were sacrificed at 1 h, 4 h, and 12 h postinjection. The heart, spleen, lung, liver, kidney, and femur were excised immediately and subsequently washed with saline three times for FITC fluorescence imaging on an in vivo imaging system (IVIS Lumina LT, PerkinElmer, Santa Clara, CA, USA). The results were quantitatively analyzed using Living Image software (PerkinElmer, USA).

After sacrifice, histological analyses of the liver, spleen, kidney, heart, and lung by H&E staining were performed to assess the in vivo toxicology 12 h after injection of Aln-FITC. An untreated SD male rat was used as the control.

### 2.7. pH-Responsive Characteristics of Aln-FITC

Aln-FITC was diluted to 1 *μ*M in PBS with pH values ranging from 2 to 14. A similar experiment was performed with 1 *μ*M Aln-FITC bound to HAp particles at pH values varying from 6 to 12 since a pH lower than 6 caused visible dissolution of HAp. The fluorescence intensity of each sample was measured with a multiplate reader. Thin bone blocks were dissected from the rat femurs as described in Section 2.6 and were also submerged in PBS with pH values from 6.8 to 7.4 and observed under a microscope (EVOS FL Auto). Excitation and emission wavelengths were adjusted according the reported maxima for Aln-FITC.

Aln-FITC was further intravenously injected into 3 male SD rats at a dose of 8 *μ*mol/kg body weight. The donor mice were sacrificed after 12 h. The femurs were excised immediately and subsequently perfused with saline for 1 min, followed by perfusion with 1x PBS at four pH values (pH 6.8, 7.0, 7.2, and 7.4). To avoid interference caused by the order of perfusion liquid or overwashing with Aln-FITC, the bones were perfused in random orders of PBS with different pH values. Then, the fluorescence intensity of Aln-FITC bound to the bone was measured on the in vivo imaging system.

### 2.8. In Vivo pH Sensing in a Metabolic Acidosis Model

Aln-FITC was intravenously injected into 3 nude mice (male, 4-6 weeks old, 8 *μ*mol/kg body weight). At 12 h postinjection, the mice were anesthetized by intraperitoneal injection of chloral hydrate and intravenously injected with 3% lactic acid to simulate metabolic acidosis. After 10 min, the mice were intravenously injected with 5% sodium bicarbonate for treatment. The fluorescence intensity was measured with the in vivo imaging system.

### 2.9. Statistical Analysis

All data are presented as the mean ± standard deviation. The difference between two groups was analyzed by unpaired Student's *t*-test, and the difference among three or more groups was analyzed by one-way ANOVA and LSD as the post hoc test. In all comparisons, *P* < 0.05 was set as the significant difference. All statistical analyses and graphs were processed in OriginPro 9.0 (OriginLab, USA).

## 3. Results and Discussion

### 3.1. Synthesis and Characterization of Aln-FITC

**Figure 1:**
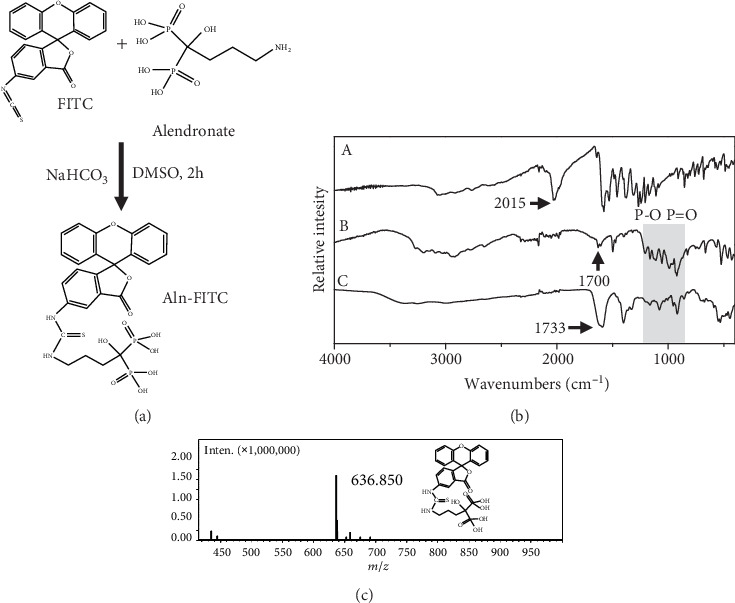
Synthesis and characterization of alendronate-FITC (Aln-FITC). (a) Schematic synthesis process of Aln-FITC. (b) FTIR spectra of FITC, alendronate, and Aln-FITC. (c) Mass spectrum of Aln-FITC.

Aln-FITC was synthesized by conjugating the pH-sensitive dye FITC with the bone-targeting ligand alendronate, as illustrated in Figure 1(a), and the final product was an orange powder. To confirm the chemical structure, the final product was analyzed by FTIR (Figure 1(b)) and MS (Figure 1(c)). As shown in Figure 1(b), free FITC displayed a peak at 2015 cm^−1^ corresponding to the isothiocyanate group, and the free alendronate showed a characteristic peak at 1700 cm^−1^ assigned to the N-H bending. After conjugation, both peaks disappeared, and a strong peak at 1733 cm^−1^ emerged, indicating the formation of thiohydantoin. At the same time, broad peaks ranging from 1300 to 750 cm^−1^ appeared in both alendronate and Aln-FITC, suggesting the presence of the remaining phosphate groups in the final product. Moreover, the MS confirmed the molecular weight of Aln-FITC as 636.85, which is consistent with the theoretical value of Aln-FITC, also indicating a successful synthesis.

The optical and fluorescent characteristics of Aln-FITC are shown in Figs S1-3. First, the absorption spectrum (Fig S1) showed a maximum absorbance peak at approximately 460 nm^−1^ in both the FITC and Aln-FITC spectra and no absorbance in the alendronate spectrum. In the fluorescence excitation spectrum (Fig S2) and the fluorescence emission spectrum (Fig S3), Aln-FITC showed a maximal excitation peak at 450-475 nm^−1^ and a maximal emission peak at approximately 525 nm^−1^, which is similar to the FITC results. Therefore, the optical and fluorescent parameters of Aln-FITC were the same as those of FITC.

### 3.2. In Vitro and In Vivo Affinity of Aln-FITC to HAp and Bone

**Figure 2:**
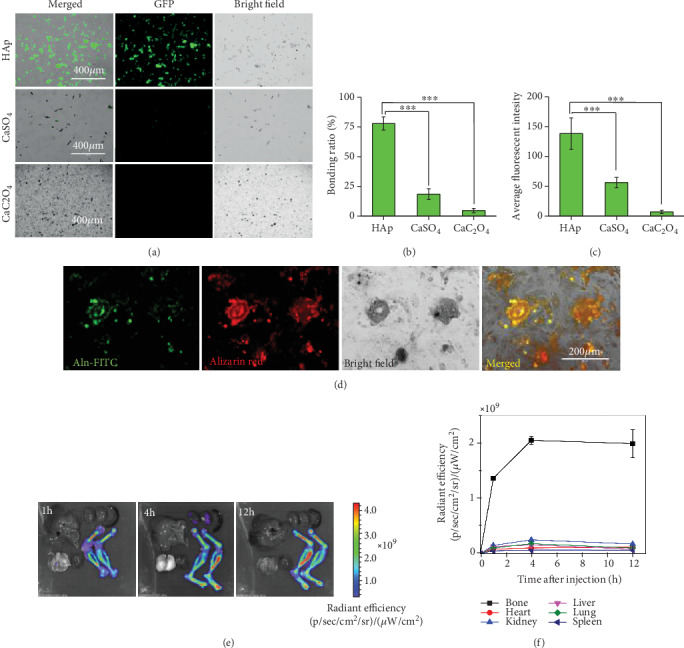
Selective affinity of Aln-FITC towards HAp and bone. (a) Fluorescence microscopy images of Aln-FITC binding to different calcium materials (HAp, CaSO_4_, and CaC_2_O_4_). Scale bar = 400 *μ*m. (b, c) Quantified analysis of the fluorescence intensity of different calcium minerals. The bonding ratio was calculated as the ratio of positive fluorescent particle numbers to total particle numbers, while the average fluorescence intensity was measured from all positive fluorescent particles. (d) Fluorescence microscopy images of osteogenic differentiated rBMSCs stained with Aln-FITC and Alizarin S. Scale bar = 200 *μ*m. (e) The in vivo biodistribution of Aln-FITC at 1 h, 4 h, and 12 h postinjection in rat hearts, livers, spleens, lungs, kidneys, and bones. (f) Quantitative analysis of the time-radiant efficiency in different organs. *N* = 3, mean ± SD.

After incubation with Aln-FITC, HAp showed the highest intensity of green fluorescence (Figure 2(a)). Quantitative analysis revealed that the bonding ratio to HAp (77.93 ± 5.50%, Figure 2(b)) was threefolds higher than that to CaSO_4_ (18.44 ± 4.59%, *P* < 0.001) and fifteenfolds higher than that to CaC_2_O_4_ (4.66 ± 1.82%, *P* < 0.001). The average fluorescence intensity (Figure 2(c)) on HAp (138.1 ± 26.3) was also higher than that on CaSO_4_ (56.1 ± 8.6, *P* < 0.001) and CaC_2_O_4_ (6.9 ± 2.8, *P* < 0.001). These results demonstrated the selective affinity of Aln-FITC towards HAp.

Fluorescence microscopy (Figure 2(d)) showed that the localization of Aln-FITC (green) basically overlapped with Alizarin S (red) in the differentiated rBMSCs, suggesting the selective affinity of Aln-FITC to calcified nodules compared with other cellular structures. This result demonstrated the selective targeting of Aln-FITC towards bone-like structures.

The in vivo targeting selectivity of Aln-FITC to the bone was also confirmed in Figure 2(e). After caudal intravenous injection of Aln-FITC, the bone showed the highest green fluorescence among the major organs, including the heart, spleen, lung, liver, and kidney. Quantitative analysis (Figure 2(f)) proved that 1 h after injection, the radiant efficiency in the bone increased to (1.36 ± 0.03) × 10^9^ units, which was almost 66.3% of the maximal radiant efficiency, suggesting a fast accumulation in the bone after only 1 h. Four hours after injection, the radiant efficiency in the bone reached its peak of (2.05 ± 0.07) × 10^9^ units. At 12 h postinjection, the radiant efficiency remained at (1.99 ± 0.26) × 10^9^ units, approximately 97.1% of the maximal radiant efficiency, indicating a long-lasting high concentration of Aln-FITC in the bone. In other organs, the highest radiant efficiency appeared in the kidney 4 h after injection, which was (2.46 ± 0.03) × 10^8^ units, suggesting that unbound Aln-FITC was excreted by the kidneys. Therefore, these results indicated that Aln-FITC is a bone-targeted sensor suitable for long-term real-time sensing.

### 3.3. Biocompatibility of Aln-FITC

The cytotoxicity test of Aln-FITC in rBMSCs (Fig S4) exhibited no marked cytotoxicity when the concentration of Aln-FITC ranged from 0.1 *μ*M to 1 *μ*M. However, the cytotoxicity became significant when the concentration increased to 5 *μ*M (0.90 ± 0.06, *P* < 0.05) and 10 *μ*M (0.86 ± 0.03, *P* < 0.01). These results indicated that Aln-FITC had good biocompatibility when the concentration was no greater than 1 *μ*M, which serves as a reference for further biological applications. The in vivo toxicology was also examined by the corresponding histological analyses of the major organs, including the liver, spleen, kidney, heart, and lung, with H&E staining (Fig S5), suggesting no apparent histological toxicology of Aln-FITC at the dose of 8 *μ*mol/kg body weight compared with the untreated control.

### 3.4. Unique pH-Responsive Characteristics of Aln-FITC on HAp and Bone

**Figure 3:**
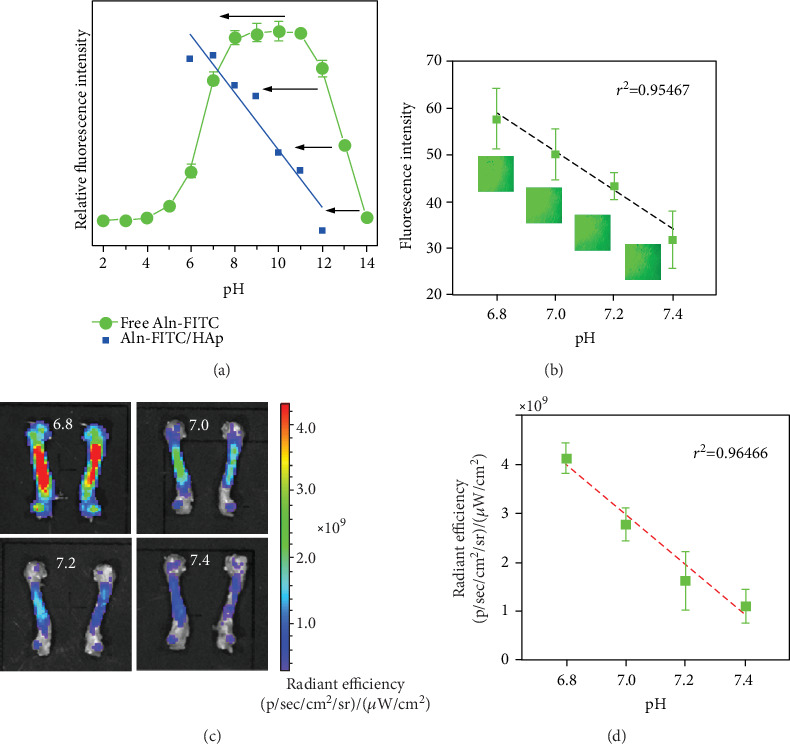
The pH-responsive characteristics of Aln-FITC after binding to HAp and bone. (a) The pH-relative fluorescence intensity curve of free Aln-FITC (green) and Aln-FITC bonding to HAp (blue). Black arrows indicate the leftward shift after binding to HAp. (b) The fluorescence intensity in femur blocks decreased when the pH increased from 6.8 to 7.4. *N* = 3, mean ± SD. Representative fluorescence microscopy images of femur blocks bonded with Aln-FITC were affiliated with the data points. Note that the bottom right corner is the thin bone at the margin and the top left corner is the central thick bone. (c) The radiant efficiency of Aln-FITC in rat perfused bones over the pH range of 6.8-7.4. (d) The calibration curve of pH-radiant efficiency showed that the radiant efficiency in perfused bone linearly decreased when the pH increased from 6.8 to 7.4. *N* = 3, mean ± SD.

The pH-responsive characteristics of Aln-FITC are shown in Figure 3. First, we studied the response curves of free Aln-FITC and Aln-FITC/HAp (Figure 3(a)). The fluorescence intensity of free Aln-FITC changed in the pH range of 2-10 in a pattern similar to many previous findings on FITC [25, 26]. However, the pH-responsive curve of Aln-FITC/HAp left-shifted compared with that of FITC, resulting in a unique decreasing trend when the pH varied from 6 to 12.

Then, we confirmed this phenomenon in bone blocks immersed in PBS at different pH values using fluorescence microscopy (Figure 3(b)). We found that the average fluorescence intensity linearly decreased (*R*^2^ = 0.95467) at pH values of 6.8 (57.61 ± 6.42), 7.0 (50.13 ± 5.30), 7.2 (43.25 ± 2.67), and 7.4 (31.78 ± 6.12), and the loss of fluorescence was more obvious in the thin part of the bone blocks than in the thick part, indicating that the change started from the bone surface.

To establish a relationship between pH change and fluorescence intensity under the in vivo imaging systems, we analyzed the radiant efficiency of Aln-FITC binding to the bone under different pH values in a perfused bone model in Figure 3(c). When the pH increased from 6.8 to 7.4, the radiant efficiency rapidly decreased. The calibration curve of the pH-radiant efficiency in the bone (Figure 3(d)) showed that the radiant efficiency was (4.2 ± 0.31) × 10^9^ units at pH 6.8 and fell to (1.1 ± 0.34) × 10^9^ units at pH 7.4, decreasing in a linear manner (*R*^2^ = 0.96466). That is, in the pH range of 6.8-7.4, the relative radiant efficiency will decrease by 24.60% when the pH increases by 0.2 units as a preliminary calibration relationship to calculate the relative pH changes in the bone.

### 3.5. Preliminary Applications in a Metabolic Acidosis Model

**Figure 4:**
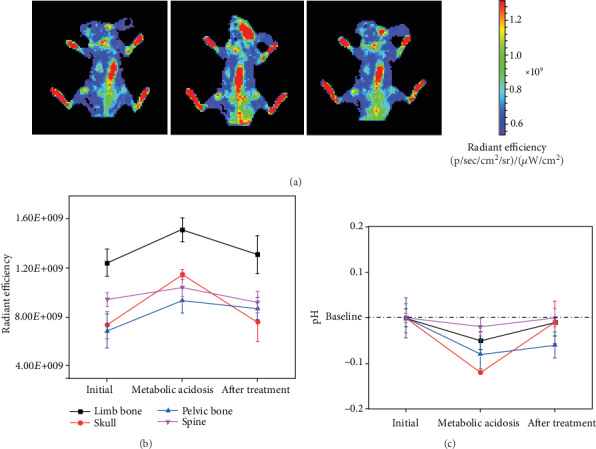
Application of Aln-FITC in sensing pH changes in a metabolic acidosis model. (a) In vivo fluorescent images of Aln-FITC in nude mice during three different states: the normal state, metabolic acidosis state, and alkaline drug treatment state. (b, c) Quantitative calculation of relative radiant efficiency and corresponding pH change of the skull (red), limbs (black), and pelvis (blue) under the three different conditions.

We further tested the ability of Aln-FITC to sense bone pH changes in a metabolic acidosis model. In this preliminary study, since rat hairs may introduce fluorescent pollution and the penetration depth of Aln-FITC is relatively low, nude mice were chosen as the model animal due to their hairless skin and thinner soft tissue. Twelve hours after injection, Aln-FITC was distributed mainly in the skull, spine, limbs, and pelvic bone, as shown in Figure 4(a). After intravenous injection of 3% lactic acid to simulate metabolic acidosis, the radiant efficiency increased by approximately 56.06% in the skull bone, 36.35% in the pelvic bone, 11.06% in the spine, and 21.62% in the limb bones. After injection of 5% sodium bicarbonate for treatment, the radiant efficiency decreased by approximately 33.68% in the skull bone, 6.9% in the pelvic bone, 11.92% in the spine, and 13.45% in the limb bones (Figure 4(b)). It has been reported that the bone has an initial pH of approximately 7.4 [2], and so we calculated the relative pH changes according to the pH-radiant efficiency relationship discovered in Section 3.4. Therefore, Aln-FITC indicated that the pH decreased by 0.12 in the skull, 0.08 in the pelvic bone, 0.02 in the spine, and 0.05 in the limb bones in the metabolic acidosis model (Figure 4(c)), and the pH value regained 0.11 units in the skull, 0.02 units in the pelvic bone, 0.02 units in the spine, and 0.04 units in the limb bones after treatment.

### 3.6. Limitations

The pH sensor in our study can be a useful tool in monitoring pH changes in basic and preclinical studies on bone physiology and diseases. However, we must point out that the maximal excitation peak of Aln-FITC is at 450-475 nm, and the maximal emission peak is at approximately 525 nm, which implies that Aln-FITC has a low penetration depth and should be used in vitro or in vivo in preclinical animal models. We also found that animals with long hairs can easily bring background pollution to the fluorescence. Therefore, future studies should introduce novel dyes with higher wavelengths to increase the penetration depth and reduce background pollution from animal hairs [27]. For human applications, fluorescent sensors should be constructed in the near infrared range, which is called the diagnostic window [28].

In our study, the fluorescence intensities in different bones in the same state were different due to the different bone masses and different Aln-FITC concentrations, which makes it difficult to determine the absolute pH value. Therefore, we chose to calculate relative pH changes according to the relative fluorescence intensity changes at a single point. Previous studies have tried to introduce a reference indicator to avoid this problem by using pH-insensitive dyes such as AuNCs and rhodamine [26, 29]. Combining a two-sensor system and bone-targeting ligand is challenging and shall be investigated in the future. In addition, compared with fluorescence intensity, novel fluorescence techniques such as fluorescein lifetime measurement [30, 31] showed better accuracy and specificity in in vivo pH sensing. Future studies should introduce fluorescence lifetime measurements to step into a broader application of bone pH sensors.

## 4. Conclusion

In this study, we developed a bone pH sensor (Aln-FITC) suitable for preclinical applications to monitor pH changes during bone healing, metabolic disease, and cancer. Aln-FITC can be applied in rodents at a biologically safe concentration, avoiding the risk of surgical wound infection in previous pH detection methods. Aln-FITC has excellent bone-targeting ability and good pH sensitivity in the pH range of 6.8-7.4, making it suitable for detecting pH changes in ex vivo or in vivo nude mouse models. However, Aln-FITC is limited by the short wavelength and quantification difficulties in the absolute pH value without an internal reference, which shall be addressed by using a two-sensor system inside the diagnostic window and novel fluorescence techniques such as fluorescence lifetime imaging in future studies.

## Data Availability

All data generated or analyzed during this study are included in this article and Supplementary Materials.

## Supplementary Materials

Supplementary Materials Figure S1: UV-vis spectra of FITC (A), Aln-FITC (B), and alendronate (C). Figure S2: excitation spectra of FITC (A), Aln-FITC (B), and alendronate (C). Figure S3: emission spectra of FITC (A), Aln-FITC (B), and alendronate (C). Figure S4: cell viability of rBMSCs treated with Aln-FITC at different concentrations (0 *μ*M, 0.1 *μ*M, 0.5 *μ*M, 1 *μ*M, 5 *μ*M, and 10 *μ*M) for 24 h, *n* = 6, mean ± SD. Figure S5: histological analysis of major organs, including the liver, spleen, kidney, heart, and lung, by H&E staining (scale bar = 50 *μ*m).
