# Supplementary figures and images for: FITC-Labeled Alendronate as an In Vivo Bone pH Sensor
Source: Biomed Res Int. 2020 May 19;2020:4012194. doi: 10.1155/2020/4012194 (PMC7256770; doi:10.1155/2020/4012194)

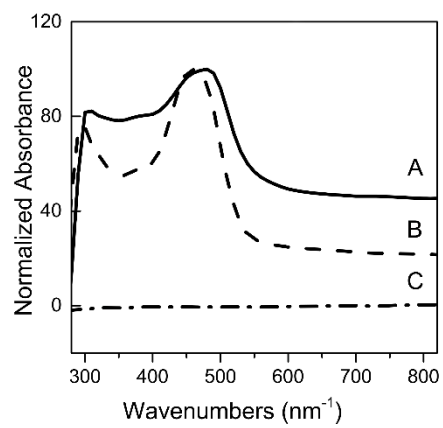

S1

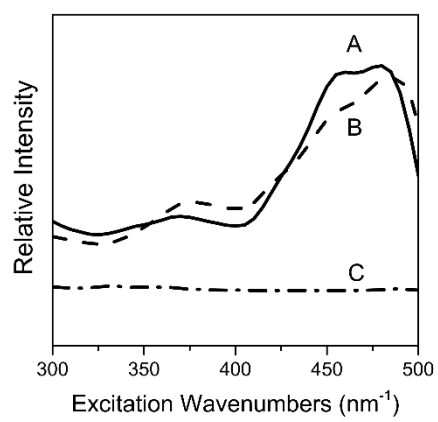

S2

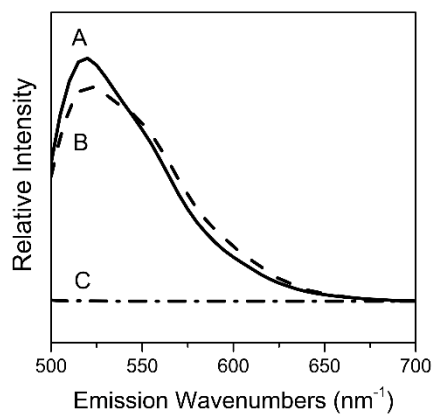

S3

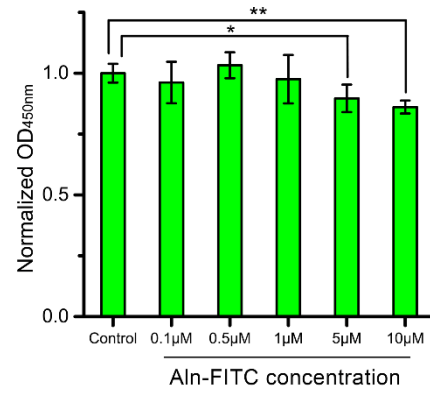

S4

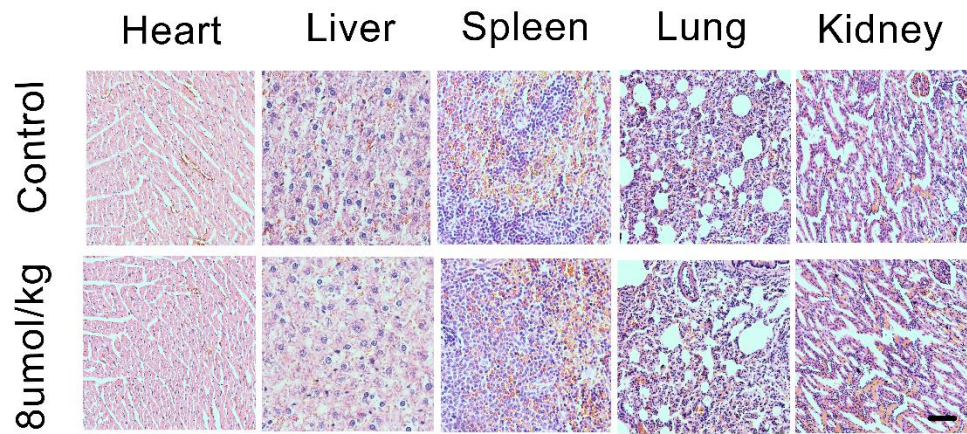

S5

Supplement: Supplementary Materials — Figure S1: UV-vis spectra of FITC (A), Aln-FITC (B), and alendronate (C). Figure S2: excitation spectra of FITC (A), Aln-FITC (B), and alendronate (C). Figure S3: emission spectra of FITC (A), Aln-FITC (B), and alendronate (C). Figure S4: cell viability of rBMSCs treated with Aln-FITC at different concentrations (0 μM, 0.1 μM, 0.5 μM, 1 μM, 5 μM, and 10 μM) for 24 h, n = 6, mean ± SD. Figure S5: histological analysis of major organs, including the liver, spleen, kidney, heart, and lung, by H&E staining (scale bar = 50 μm). [file 4012194.f1.pdf]
